# Lens apoptosis in the *Astyanax* blind cavefish is not triggered by its small size or defects in morphogenesis

**DOI:** 10.1371/journal.pone.0172302

**Published:** 2017-02-24

**Authors:** Hélène Hinaux, Gaëlle Recher, Alexandre Alié, Laurent Legendre, Maryline Blin, Sylvie Rétaux

**Affiliations:** 1 DECA group, Paris-Saclay Institute of Neuroscience, UMR9197, CNRS, Université Paris Sud, Université Paris-Saclay, Avenue de la terrasse, Gif-sur-Yvette, France; 2 Plateforme BioEmergence, USR3695, CNRS, Université Paris-Saclay, Avenue de la terrasse, Gif-sur-Yvette, France; 3 UMS AMAGEN (UMS 3504 CNRS / UMS 1374 INRA), CNRS, INRA, Université Paris-Saclay, Avenue de la terrasse, Gif-sur-Yvette, France; Laboratoire Arago, FRANCE

## Abstract

Blindness is a convergent trait in many cave animals of various phyla. *Astyanax mexicanus* cavefish is one of the best studied cave animals; however the mechanisms underlying eye degeneration in this species are not yet completely understood. The lens seems to play a central role, but only relatively late differentiation defects have been implicated in the cavefish lens apoptosis phenotype so far. Here, we used genetic crosses between *Astyanax* cavefish and surface fish to confirm that during development, lens size is independent of retina size. We then investigated whether the small size of the cavefish lens could directly cause cell death. Laser ablation experiments of lens placode cells in surface fish embryos showed that a small lens size is not sufficient to trigger lens apoptosis. We further examined potential lens morphogenesis defects through classical histology and live-imaging microscopy. From lens placode to lens ball, we found that lens invagination and formation of the lens epithelium and fiber cells occur normally in cavefish. We conclude that the main and deleterious defect in the *Astyanax* cavefish lens must concern the molecular control of lens cell function.

## Introduction

Cave animals show very consistently a blind phenotype [1]. This convergent loss of a sensory modality in various phyla is striking. However it is unknown whether similar mechanisms underlie eye loss in different cave species, as they remain unknown or even unexplored in most cases. The blind *Astyanax mexicanus* cavefish (CF) is one of the best studied cave animals, in particular because in this species, there are also eyed river-dwelling fishes (called surface fish, SF), which can be used for comparative studies [2, 3]. Furthermore, this species comprises 29 different cave populations, some of which evolved independently [4], which allows the study of convergent evolution. The degeneration of the eye in the *Astyanax* cavefish is one of the most studied blind phenotypes associated with troglomorphic life, however the picture is still not entirely clear. *Astyanax* cavefish embryos first develop an eye, with a correctly formed eye cup (the future retina, *i*.*e*., the neural part of the eye) and lens (derived from a placode, a non-neural ectodermal thickening) [5, 6]. The lens enters apoptosis after the embryos have hatched, at about 40–42 hours post fertilization (hpf), and the retina and the rest of the eye later degenerate [7], giving rise to eyeless adult cavefish. The triggering event of this degenerative process is lens apoptosis, an event that is autonomous to the lens, as shown by lens transplantation experiments between SF and CF embryos [8, 9].

The lens of the cavefish presents several described defects: it is smaller than the SF lens [5], partly because of early signalling modifications at the end of gastrulation [10]; it doesn’t express correctly the chaperone Hsp90α [11]; it also does not express a number of crystallins [12–14], which are lens differentiation genes. One of the crystallins absent from the cavefish lens, *cryaa*, has been shown to be involved in lens apoptosis [13, 15]. Nevertheless, several QTLs influence eye size [16, 17], and eye loss is most likely not due to a single defect. Here, we set out to examine several possible cellular mechanisms that may explain the apoptotic phenotype of the *Astyanax* cavefish lens.

A hypothesis that has not been tested yet is whether the small size of the cavefish lens could directly induce cell death.

Morphogenesis of the *Astyanax* cavefish lens has not been explored extensively either thus far. The vertebrate lens develops from a placode, a thickening of the non-neural ectoderm, and transitions from this 2D structure to a spherical one composed of different cell-types (the internal fibre cells and the external lens ectodermal cells) occur through morphogenetic events. In zebrafish (*Danio rerio*), these steps have been described in detail: thickening of the placodal ectoderm, invagination of the lens mass and detachment from the surface ectoderm, reorganization of the lens ectoderm cells into one cell layer and elongation of the lens fiber cells [18–20]. Defects in lens morphogenesis can lead to apoptosis: transgenic mice expressing a mutant version of the SV40 large T antigen in the lens have a defect in fiber cell morphogenesis, leading to apoptosis and severe microphtalmia [21]. Other mutant mice show defects in lens morphogenesis which result in microphtalmia [22–24]. Such defects are also known to cause ocular anomalies in humans, as for instance in Peters anomaly which results from the incomplete separation of the lens from the surface ectoderm and the persistence of the lens stalk [25].

We therefore decided to explore in more details *Astyanax* cavefish lens development. We specifically addressed the potential link between its small size and apoptosis, and we analysed its morphogenesis.

## Materials and methods

### Animals

Laboratory stocks of *A*. *mexicanus* surface fish, Pachón cave fish and Molino cave fish were obtained in 2004 from the Jeffery laboratory at the University of Maryland, College Park, MD. They had been lab-raised for some generations (except for Molino fish that are wild animals), and surface fish had initially been collected in San Solomon Spring, Balmorhea State Park, Texas. In our facility, they were maintained and bred at 23°C (Pachón and Molino) and 26°C (surface) on a 12:12 hours light/dark cycle in tap water. Surface and Pachón cavefish embryos were collected after natural spawning, staged according to the developmental staging table [26] and fixed at various stages in 4% paraformaldehyde (PFA). After progressive dehydration in methanol, they were stored at –20°C.

Animals are treated according to the French and European regulations for handling of animals in research. SR’s authorization for use of animals in research is number 91–116. Paris Centre-Sud Ethic Committee approved the study and the authorization number is 2012–0052.

### Eye and lens measurements

The hybrid larvae were obtained by *in vitro* fertilization of SF or Pachón eggs by sperm of SF, Pachón or Molino fish [27]. They were bred at 23°C, and photographed at 36hpf under an Olympus SZX16 stereomicroscope. Eyeball and lens measurements were performed on the pictures using ImageJ software.

### In situ hybridizations

cDNAs were amplified by PCR from pCMV-Sport6 plasmids picked from our cDNA library [28] and digoxygenin-labeled riboprobes were synthesised from PCR templates. A protocol for automated whole-mount *in situ* hybridization (Intavis) was performed. Briefly, embryos were progressively re-hydrated, permeabilized by proteinase K (Sigma) treatment before being incubated over night at 68° in hybridization buffer containing the appropriate probe. After stringent washes, the hybridized probes were detected by immunohistochemistry using an alkaline phosphatase-conjugated antibody against digoxygenin (Roche) and a NBT/BCIP chromogenic substrate (Roche). After staining, embryos were photographed *in toto*, always in the same orientation, under a Nikon AZ100 stereomicroscope using agarose wells.

### Laser ablation

SF embryos were collected at 6hpf and incubated in 60 μM Bodipy 505/515 (D3921, Invitrogen) diluted in embryo medium (EM) for 1 hour after chorion removal. They were then washed in EM, and grown at 23°C until 12hpf. They were mounted laterally in agarose wells (1% in EM, SIGMA), one optic vesicle facing the objective.

Images were acquired at the BioEmergences facility (http://bioemergences.iscpif.fr) with a custom made two-photon microscope based on a Leica microscope (DM 6000 stand, and SP5 scan head) coupled with a MAITAI Spectra Physics femtosecond laser. A high numerical aperture objective (Leica, HCX APO L20x/1.00 W) was used to focus the laser to the sample and to collect Bodipy fluorescence. Light was detected with a Leica Hybrid detector (detection filter 525center/50width).

Laser non-invasive imaging was performed just before photo-ablation to localize the large cells in the surface ectoderm adjacent to the optic vesicle (putative lens precursor cells), as well as immediately after and 24h after photo-ablation to measure the size of the lens on the ablated and control sides. Laser was tuned at 900 nm or 980 nm with a pixel size comprised between 0.3 μm and 1 μm depending on experiments (512*512 pixels), 3 frames averaged (frame averaging mode), 400 Hz scan speed.

Photo-ablation [29, 30] was performed on the putative lens precursor cells. Because these are located at the border of the eye vesicle, laser intensity was only weakly attenuated due to scattering in depth. But the variability of the samples implied to adapt laser ablation conditions to each sample. The laser was either used at 900 nm or 860 nm (to increase the overall deposition of energy), a 25 zoom factor was applied (pixel width was 0.06 μm) and image was averaged 8 times (line averaging mode). Depending on experiments, image size was 1024*512 or 512*512, and scanning speed could be decreased down to 200 Hz. When deleterious effects started to be visible (cavitation bubbles formation), the ablation step was immediately stopped. The sample was scanned again at low power and low magnification to attest the efficiency and the reproducibility of the ablation (similar number of cells ablated and no obvious surrounding damage).

After ablation, embryos were grown in EM with PTU (N-Phenylthiourea, P7629, SIGMA, 1/1000 of stock solution at 3% in Ethanol) overnight at 23°C, incubated again 1 hour in Bodipy 505/515, washed in EM, and their eyes were imaged upon non-invasive two-photon excitation to assess the size of the lens and the retina. They were then fixed in PFA 4% at 60hpf and processed for TUNEL assay (Promega).

### 4D imaging

*Astyanax* eggs were obtained by *in vitro* fertilization [27], and injected at one cell stage with a mix of mRNAs at 100 ng/μL, encoding H2B-mCherry and Ras-EGFP. When they reached 6 hpf, embryos were sorted to remove any abnormal phenotype and to choose the most fluorescent ones. Their chorion was removed, and they were mounted in custom-made Teflon molds at 9–10 hpf and maintained with low melting agarose (0.4%, low melt agarose, 6351.5, ROTH), future head facing the objective, in a medium containing PTU (1/1000 of stock solution at 3% in Ethanol) and tricaine (A5040, SIGMA, 1/10 of stock solution at 0.4% in water).

They were then imaged upon two photon excitation on upright stands (Leica DM6000 and DM5000 stands with SP5 scan head, and Zeiss Axio Examiner stand with LSM780 scan head and GaAsP spectral detection; Lasers for Leicas: t-pulse (Amplitude System) at 1030 nm, and Maitai (Spectraphysics) tuned at 980 nm; Laser for Zeiss: Chameleon XR (Coherent) tuned at 810 nm).

Acquisition lasted for 15–20 hours while temperature was maintained at 23°C (OKO-Lab system). Beam was focused on the samples with the following objectives: Leica 1.0 NA 20X W (HCX APO), Olympus 0.95 NA 20W W (XLUMPlanFluo) and Nikon 1.1 NA 25X (CFI Apo LWD). Fluorescence was epicollected and directed to the detectors (Hybrid units for the Leicas, and GaAsP spectral module for the Zeiss) and selected with bandpass filters (Leicas: 525/50 nm (EGFP), 610/75 nm or 585/40 nm (mCherry)) and tunable filters for the Zeiss). Excitation 980 or 1030 nm 42-88mW; scan speed 300–1200 Hz; frame average 1–2; zoom 1–1.5; 512*512 pixel at 0.9–1.2 μm wide. A full z-stack was compiled in 2–4 minutes. After imaging, embryos were removed from the molds and checked for damage. After acquisition, raw images were converted into VTK format and uploaded on the BioEmergences workflow (http://bioemergences.iscpif.fr/workflow/) to be stored and processed (developed by BioEmergences, UPS 3674) [31].

## Results

### Insights from surface fish × cavefish genetic crosses

**Fig 1:**
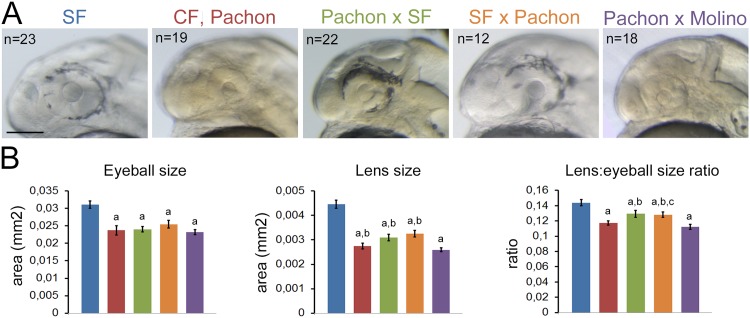
Lens size and eye size are controlled by independent and early mechanisms. **A**, Photographs of the head of 36hpf individuals from SF, CF and F1 hybrids as indicated. For hybrids, the first name given is the origin of the female used in the cross. The number of larvae examined is indicated. Scale bar: 100μm. **B**, Histograms showing lens and eyeball area measurements and lens:eyeball ratios in 36hpf larvae. Color code as in A: SF, blue; Pachón, red; Pachón × SF, green; SF × Pachón, orange; Pachón × Molino, purple. For crosses, the female parent is given first. Data are mean±s.e.m. Significant differences are indicated: a, different from SF (p<0.01 to p<0.0001); b, different from Pachón × Molino (p<0.05 to p<0.001); c, different from Pachón (p<0.05), Mann Whitney tests.

The eye of vertebrates has two main components, the neural retina and the lens. In *Astyanax* cavefish, these two structures are reduced in size during development [5, 6, 32]. The small size of the cavefish lens could thus be an indirect consequence of the small retina, as the presumptive lens receives inductive signals from the retina [33–35]. This scenario is unlikely because the presumptive lens placode territory is already reduced in cavefish at the end of gastrulation, before inductive events take place [10]. Alternatively the reduced size of the CF lens could be directly genetically encoded. Genetic evidence from eye anatomical analyses or QTL studies using adult F2 hybrid individuals after SF×CF crosses suggests that the retina and the lens structures are subjected to separate genetic control and that the size of these two eye components are controlled by different loci [36–38]. Here, to further explore an early developmental origin in the lens size differences between CF and SF and to rule out a possible dependence on the retinal size, we measured lens and eyeball sizes in 36hpf F1 hybrid larvae resulting from various crosses between SF and two different populations of CF originating from the Pachón and Molino caves (Fig 1A). At such an early stage, shortly after hatching [26] the lens size, the eyeball size, and the lens/eyeball size ratio vary among the different F1 larvae (Fig 1B). In F1 hybrids, the proportion of eyeball size occupied by the lens is higher than in Pachón embryos, although the size of their entire eyeball is identical. This suggests that the reductions in lens size and the reduction in eye size in cavefish are controlled by 1) independent and 2) early embryonic mechanisms.

### Reduced lens size in cavefish: A direct role in triggering lens apoptosis?

The size of the lens is apparently controlled independently from the retina [36–38](and present data). The size in itself could therefore represent the endogenous defect responsible for triggering apoptosis in the lens. We decided to test whether CF lens cells would not be numerous enough to differentiate properly.

**Fig 2:**
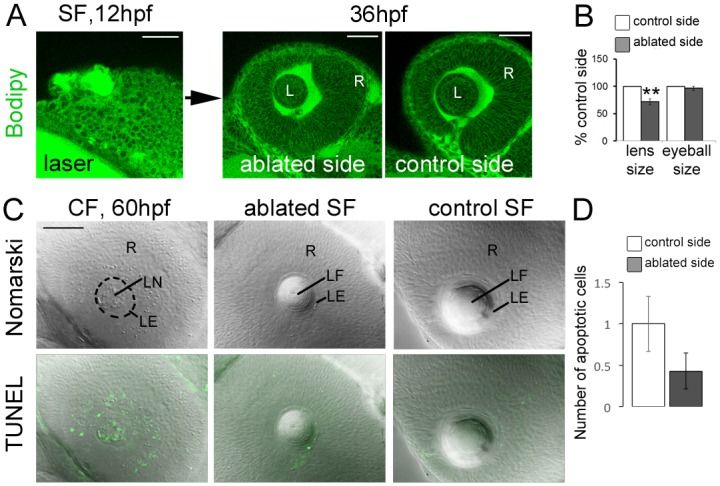
Reducing lens size in SF does not cause apoptosis. **A**, Non-invasive two-photon imaging showing laser ablation of the lens placode in SF embryos at 12hpf and the resulting anatomy of the eye 24 hours later. Embryos were stained with Bodipy 505/515 which labels membranes. L, lens; R, retina. Scale bars, 50μm. **B**, Histogram showing quantification of lens and eye size area after ablation and in the contra-lateral un-operated side. Data are mean±s.e.m. Significant differences are indicated: **p = 0.0051; Wilcoxon test. **C**, Photographs of the eyes of 60hpf larvae in the indicated conditions, after TUNEL staining (green fluorescence). Top images show Nomarski images to assess anatomy and cytoarchitectony, and bottom images show the merged TUNEL fluorescent staining. LF, lens fibers; LE, lens epithelium; LN, lens nucleus; R, retina. Scale bar, 50μm. **D**, Quantification of the TUNEL positive cells per SF lens on the control and laser-ablated side (n = 7).

To test this hypothesis, we performed unilateral laser ablation of lens placode cells at 12-14hpf on SF embryos, to mimic the small lens component of CF (Fig 2A). One day later, at 36hpf, this resulted in a reduction in lens size ranging from -15% to -70%, depending on the extent of ablation on the laser-treated side. Importantly, laser ablation specifically affected the size of the lens, without affecting the size of the retina part of the eye (n = 17, Fig 2A and 2B). Ablated SF larvae were grown up to 60hpf, a stage where apoptosis is massive in the lens (and also in the retina) in Pachón CF (Fig 2C, left). However in laser-treated SF, while the lens was still reduced in size at 60hpf, the morphology and cytoarchitectony of the lens epithelium, lens fiber cells and retina were normal, and apoptotic cells were as rare as in the non-operated side, even in the most severely ablated embryos (Fig 2C, middle and right, Fig 2D). These results suggest that a reduction in the number of lens cells in CF is probably not sufficient to cause their apoptosis.

### Lens morphogenesis

Since the size of the lens is not directly responsible for the apoptosis phenotype in cavefish, we decided to investigate whether the morphogenesis of the lens could be defective in Pachón cavefish and explain the degeneration process.

**Fig 3:**
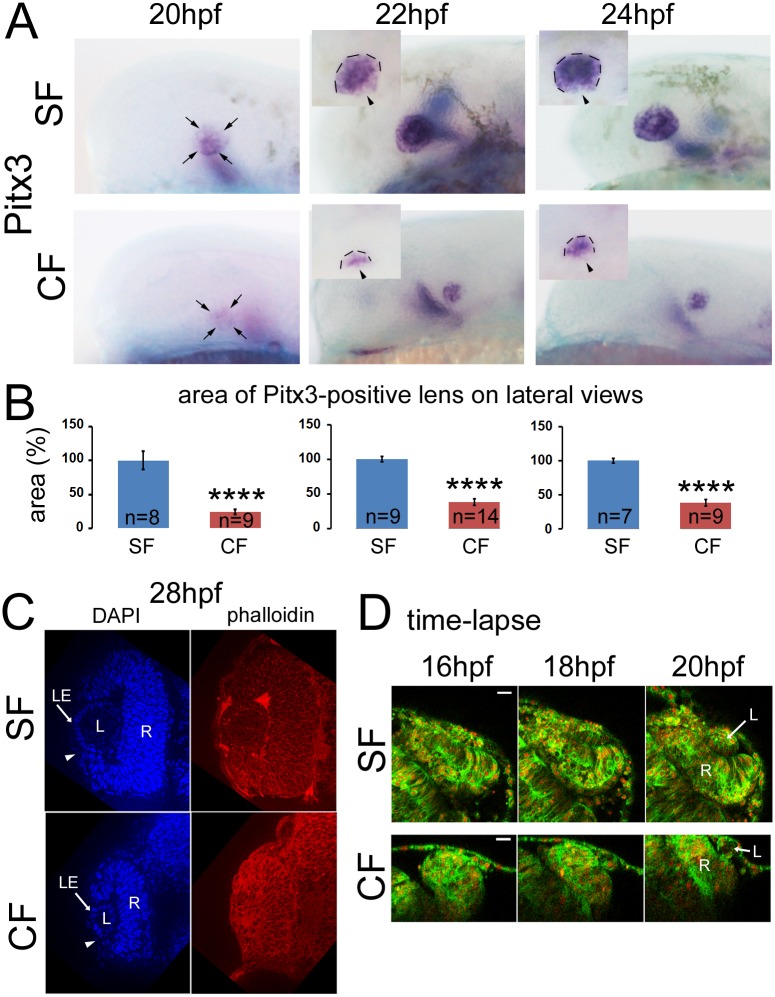
Lens morphogenesis occurs correctly in cavefish. **A**, Time course of *Pitx3* expression in the lens between 20hpf and 24hpf in SF (top) and CF (low) embryos, on lateral views. The insets correspond to photographs taken in dorsal view. An additional expression domain of *Pitx3* inside the brain is also visible (out of focus on the pictures). Pigment cells around the eye are visible on SF but not CF. Arrowheads show the external part of the lens, facing towards the surface ectoderm. **B**, Corresponding quantification of lens size (measured on lateral views) between 20hpf and 24hpf in SF (blue) and CF (red). Data are mean±s.e.m. Significant differences are indicated: ***p<0.001; ****p<0.0001), Mann Whitney tests. **C**, Sections through the eye of SF (top) and CF (low) 28hpf larvae, stained with DAPI (left) and phalloidin (right). **D**, Time-lapse recording of lens morphogenesis in SF (top) and CF (low) embryos injected with H2B-mCherry (nuclear red labelling) and Ras-GFP (membrane green labelling) and imaged with a two-photon microscope, between 16 and 20hpf. Scale bar 25μm.

From 20hpf onwards, the lens mass could be clearly identified using *Pitx3* expression (Fig 3A). At these stages, *Pitx3* is expressed throughout the lens mass in both SF and CF [10]; therefore *Pitx3* expression area is a good proxy for lens size. From 20hpf to 24hpf, the *Pitx3*-positive lens area was much smaller in CF than in SF embryos (Fig 3B), confirming the smaller size of the CF lens. The timing of the formation of the lens mass and its delamination from the surface ectoderm, visible on dorsal views (insets in Fig 3A), was comparable in SF and CF embryos. At 28hpf, lens sections labelled with DAPI and phalloidin showed that invagination was completed in SF and CF at this stage, with surface ectoderm covering the lens mass (Fig 3C). Moreover, the formation of the lens epithelium and the first appearance of lens fibers were also visible in both morphs, despite the much smaller size of the lens in CF (Fig 3C).

The techniques used above however involve fixation which can deform the tissue. To get a better idea of the dynamics of morphogenesis in both morphs, we turned to live imaging with two-photon microscopy. We imaged *Astyanax* embryos injected with mRNAs encoding H2B-mCherry and Ras-GFP, which respectively label nuclei and membranes (Fig 3D). Technical difficulties inherent to the cavefish embryonic tissue did not allow us to get images of the same quality for CF as for SF. Despite these difficulties, the movies showed a similar timing and identical steps for the invagination of the lens placode in SF and CF: at 16hpf, the lens placode is clearly recognizable as a thickened ectoderm in both morphs (Fig 3D, left panels): both in SF and CF future lens cells form a distinct group of epithelial cells below the surface ectoderm. At 18hpf, these cells start to invaginate, they are not in a single layer anymore but form a lens mass that is still attached to the surface ectoderm (Fig 3D, middle). At 20hpf finally, the lens mass, now spherical, is fully invaginated (Fig 3D, right). This suggests that lens morphogenesis occurs normally in cavefish, and thus is not likely involved in the lens apoptosis and eye degeneration processes.

## Discussion

*Astyanax mexicanus* cavefish is one of the most extensively studied cave animals, and the mechanisms underlying its degenerated eye phenotype start to be understood. The lens has long been identified as the main defective eye tissue in cavefish: transplanting a cavefish lens into a surface fish optic cup is enough to trigger apoptosis in the surface fish eye [9]. Differentiation anomalies described in the cavefish lens could explain why it enters apoptosis [11–15]. Here we tested whether other mechanisms could participate in the eye degeneration process.

We investigated whether the small size of the cavefish lens, or its morphogenesis, could participate in lens apoptosis. We first performed crosses and measured lens and retina size on hybrid larvae to ascertain that lens size is controlled independently of retinal size, and from an early stage on (Fig 1). QTL studies already suggested that the size of these two eye components is regulated independently [36, 38], but eye measurements were performed in adults, and did not investigate early eye development events. Our results are also in agreement with histological studies showing that the degree of differentiation of the retina and lens are uncorrelated in *Astyanax* cavefish and hybrid larvae [36, 38–40]: large lenses are in some individuals combined with undifferentiated retinas, or well-developed retinas with rudimentary lenses. Moreover, and from a structural point of view, lens variations and retina variations manifest themselves independently, further suggesting that separate genetic controls act in the development of the various parts of the eye [37].

We have then directly tested whether the reduced number of cells could be causal in the advent of later lens defect and apoptosis. Interestingly, the results of laser ablation experiments performed in SF show that lens placode size is not determinant for its proper development. Even larvae with very small lenses did not show any sign of lens apoptosis. This negative result is however interesting, because it rules out the simple and attractive possibility that the cavefish lens cells are not numerous enough to differentiate properly. Moreover and to our knowledge this possibility had never been tested before. It remains possible that the small size of the cavefish lens participates in triggering cell death, but only in absence of the anti-apoptotic crystallin *cryaa* [41]. In laser-ablated SF (unlike CF), this crystallin is correctly expressed and could protect lens cells from cell death.

We also investigated the possibility that lens morphogenesis defects contribute to the cavefish lens apoptosis. To this end we turned to histology and live imaging. Invagination seems to proceed in a similar way and speed in surface and cave morphs, from 16 to 20hpf, as deduced from *in vivo* imaging. Of note, *Pitx3 in situ* hybridizations suggest a delayed timing compared to live imaging results, but we relied on the latter for determination of exact timing of lens invagination. Alternatively, pictures of whole-mount fixed embryos can be accurately used to compare the two morphs for other aspects, and they also show synchronous onset and expression dynamics of *Pitx3* expression in SF and CF. However they lack indications about the precise timing of the morphogenetic events. Finally histological sections confirmed the identical organization of the SF and CF lenses at the end of the morphogenesis period, including an outer lens epithelium and an inner lens fiber compartment.

The timing of invagination of the lens seems to be slightly faster in *Astyanax* than in zebrafish: in this model species, invagination starts around 17hpf, and is completed only at 24hpf [18, 19] *vs* 20hpf for *Astyanax mexicanus*. This is not entirely surprising as hatching occurs also earlier in *Astyanax* [26], as well as many developmental events already studied: end of somitogenesis, development of the otic vesicle, first heart beat [26], or first serotonergic neurons [42].

In conclusion, the lack of any clear morphogenetic defect in the cavefish lens suggests that apoptosis is triggered by other defects. The lens small size not being involved either, lens differentiation defects are so far the only ones that seem to participate in the cavefish eye degeneration [13, 15]. Yet these late defects probably depend on some of the early differences in the signaling environment between the two morphs, notably the *Sonic Hedgehog (Shh)* enlarged expression pattern in CF embryos at the end of gastrulation, given that SF embryos injected with *Shh* mRNA exhibit some apoptosis in their lens [43]. The targets of this early Hedgehog hyper-signaling in the lens remain elusive and will deserve further investigations.
